# Efficacy Assessment of Phage Therapy in Treating *Staphylococcus aureus*-Induced Mastitis in Mice

**DOI:** 10.3390/v14030620

**Published:** 2022-03-16

**Authors:** Fei Teng, Xiaoyu Xiong, Songsong Zhang, Guiwei Li, Ruichong Wang, Lanlan Zhang, Xiaona Wang, Han Zhou, Jiaxuan Li, Yijing Li, Yanping Jiang, Wen Cui, Lijie Tang, Li Wang, Xinyuan Qiao

**Affiliations:** 1 Heilongjiang Key Laboratory for Animal Disease Control and Pharmaceutical Development, Department of Preventive, Veterinary Medicine, College of Veterinary Medicine, Northeast Agricultural University, Harbin 150038, China; teng1085579571@163.com (F.T.); sum670238789@163.com (X.X.); s15130036557@126.com (S.Z.); xiaonawang0319@163.com (X.W.); zhouhan9659@163.com (H.Z.); lijiaxuan.1993@163.com (J.L.); yijingli@163.com (Y.L.); jiangyanping2017@126.com (Y.J.); cuiwen_200@163.com (W.C.); tanglijie@neau.edu.cn (L.T.); 2 Branch of Animal Husbandry and Veterinary of Heilongjiang Academy of Agricultural Sciences, Qiqihar 161000, China; hljslgyxh@163.com; 3 Department for Radiological Protection, Heilongjiang Province Center for Disease Control and Prevention, Harbin 150030, China; mice4@126.com; 4 Promotion Demonstration Department of Heilongjiang Fishery Technology Extension Station, Harbin 150030, China; zlllgw@163.com

**Keywords:** *Staphylococcus aureus*, mastitis, drug-resistant, phage

## Abstract

The primary aim of this study was to evaluate the efficacy of phage against mastitis induced by drug-resistant *S. aureus* in a mouse model. In this study, five *S. aureus* phages—4086-1, 4086-2, 4086-3, 4086-4, and 4086-6—were isolated from milk samples secreted by mastitis cows. Transmission electron microscopy showed that all the five phages had icosahedral heads and short non-contractile tails, which are typical characteristics of the family Podoviridae. All these phages were species-specific against *S. aureus*. The one-step growth curve showed a short latency period (10–20 min) and high burst size (up to 400 PFU/infected cell). To evaluate the effectiveness of the phage 4086-1 in the treatment against mastitis, a mouse model of mastitis was challenged with drug-resistant *S. aureus*. The results showed the proliferation of *S. aureus* in the mammary glands was significantly inhibited after treating by phage 4086-1. The concentrations of TNF-α and IL-6 decreased significantly, which demonstrated the phages could effectively alleviate the inflammatory responses. Furthermore, the histopathological analysis showed that inflammatory infiltration in the mammary glands was significantly reduced. These results demonstrate that phage may be a promising alternative therapy against mastitis caused by drug-resistant *S. aureus*.

## 1. Introduction

Bovine mastitis is a major cause of economic losses in the dairy industry as well as a public health concern [1]. Mastitis may be caused by several microorganisms, while *S. aureus* is one of the most frequent causative agents. In some countries, the prevalence is even as high as 50% [2]. *S. aureus* is a gram-positive bacterium involved in various infectious diseases of humans and animals, which is difficult to treat [3,4]. *S. aureus* infections can lead to a variety of symptoms with severity ranging from mild to life-threatening [5]. Currently, diseases caused by *S. aureus* are widely treated with antibiotics [6].

Fleming’s discovery of penicillin in the 1940s pioneered the era of antibiotics for the treatment of infections [7]. With the widespread use of penicillin, infectious diseases caused by *S. aureus* were well controlled at the time. However, many drug-resistant bacteria, such as methicillin-resistant *S. aureus* (MRSA) and vancomycin-resistant enterococci, have emerged [8]. In recent decades, MRSA has rapidly become the most common drug-resistant pathogen discovered worldwide [9,10]. In addition, MRSA have a strong ability for biofilm formation, which can further complicate eradication by increasing resistance to antimicrobial agents and the host immune response. Currently, vancomycin is the most effective antibiotic for treating MRSA infection and can inhibit the synthesis of cell walls. However, the sensitivity of MRSA to vancomycin is gradually decreasing, and the number of multidrug-resistant *S. aureus* strains is increasing sharply. Therefore, finding an effective treatment for *S. aureus* infection is an urgent problem that needs to be solved.

Phages are viruses that infect bacteria and are host specific. In recent years, antibiotic therapy for bacterial infections has been facing unprecedented challenges due to rising rates of drug-resistant infections worldwide [11]. The emergence of a series of drug-resistant strains has led to increased attention towards phage research [12]. Some phages have been proven to be effective in treating bacterial infections in animals. Compared with antibiotics, high specificity and fast self-proliferation are the unique advantages of phages [13]. Moreover, phages must recognize and bind to specific bacterial receptors so they cannot infect human or animal cells. Therefore, the side effects of phage therapy applied to humans and animals are considered to be minimal [14]. In this regard, phage therapy may be an alternative approach to the treatment of bacterial infections in animals, which has already been successfully demonstrated in several animal models [15,16].

Antibiotic resistance greatly challenges clinical treatment of Bovine mastitis. Therefore, new strategies to control *S. aureus* infection are required in the dairy industry. The primary aim of this study was to evaluate the efficacy of phage against mastitis induced by drug-resistant *S. aureus* in a mouse model. In the present study, five strains of *S. aureus* phages were isolated from milk samples secreted by mastitis cows and their biological characteristics were identified, which provided experimental data required for in vivo study application. Furthermore, the efficacy of phage against mastitis compared to a commercial antibiotic was evaluated in mice with induced mastitis, which provides preclinical evidence for the feasibility of phage therapy against mastitis.

## 2. Materials and Methods

### 2.1. Bacterial Strains

*S. aureus* CVCC 546, *Staphylococcus gallinarum* CVCC 528, and *Escherichia coli* CVCC 10,141 were purchased from the China Veterinary Microbial Culture Management Center (CVCC, Beijing, China). *S. aureus* 4086, *S. aureus* Ben, *S. aureus* ATCC 43,300, *Staphylococcus xylosus* 17, *Micrococcus luteus* 26,003, *Staphylococcus saparophytics* 17, *Staphylococcus saparophytics* E4, *Staphylococcus* *saparophytics* X4, *Staphylococcus haemolyticus* 13, *Enterococcus faecalis* 13, *Bacillus subtilis*, and *Pasteurella* P-1059 were all preserved in our laboratory.

### 2.2. Phage Isolation and Culture Conditions

The milk samples secreted by cows with mastitis were collected from cattle farms in Heilongjiang Province. *S. aureus* CVCC 546 was used as the indicator strain for phage isolation from milk samples. The milk samples were centrifuged at 6500× *g* for 20 min. The supernatants were collected and filtered with a 0.22-μm pore size filter [17,18]. Each sample filtrate (0.5 mL) and 0.2 mL of fresh culture of the indicator strain (10^8^ colony-forming units [CFU]/mL) was added to 7 mL of LB semi-solid medium and incubated at 37 °C. Single plaques were selected and inoculated in 5 mL of LB broth, and 100 μL indicator bacteria were added and cultured overnight at 37 °C at 200 rpm. The phages were purified by five cycles of resuspension, dilution, and replating individual lysed plaque, respectively. Phage enumeration (plaque-forming units (PFU)/mL) was performed using the double-layer plaque titration method [19], and the operation was repeated three times.

### 2.3. Transmission Electron Microscopy

The morphology particles of phages were examined by transmission electron microscopy (TEM). A total of 10 μL of purified phage particles (1 × 10^8^ PFU/mL) were dropped onto a copper acid grid for about 10 min and stained with 2% (*w*/*v*) phosphotungstic acid. After drying, the phages were observed under an H-7650 TEM (Hitachi Co., Tokyo, Japan).

### 2.4. Spot Test

*S. aureus* CCVC 546, *S. aureus* 4086, *S. aureus* Ben, *S. aureus* ATCC 43,300, *S. xylosus* 17, *M. luteus* 26,003, *S. saparophytics* 17, *S. saparophytics* E4, *S. saparophytics* X4, *S. haemolyticus* 13, *E. faecalis* 13, *B. subtilis*, *Pasteurella* p 1059, *S. gallinarum* CVCC 528, *E. coli* BL21, *E. coli* DH 5 α, and *E. coli* CVCC 10,141 were used to test the infectivity of the isolated phages. Then, 200 μL of fresh bacterial culture was spread on an LB agar plate. Then, 4 μL of phage cultures (1 × 10^9^ PFU/mL) were added to the center of the plate respectively after it was completely dried. After incubation at 37 °C for 12 h, the *S. aureus* culture without phage was incubated at 37 °C overnight as the control.

### 2.5. Optimal Multiplicity of Infection

The host bacteria (*S. aureus* CVCC 546) were cultured in LB medium at 37 °C with shaking at 200 rpm until the growth reached an exponential phase (optical density at 600 nm [OD_600_] = 0.6). *S. aureus* cultures were then diluted 1:100 in fresh LB medium and cultured in a shaking incubator at 37 °C. Then, the corresponding phages were added into the host cultures according to the multiplicity of infection (MOI) ratios of 0.001, 0.01, 0.1, 1, and 10. All samples were cultured in LB medium at 37 °C for 4 h. The phage lysates were centrifuged at 12,000× *g* for 4 min.

The phage titers were determined using the double-layer plate method, and the operation was repeated three times. The optimal MOI was determined when the phage titer reached its highest value. The assay was performed in triplicate.

### 2.6. One-Step Growth Curve

*S. aureus* CVCC 546 was inoculated at a rate of 2% into 20 mL LB broth and cultured at 37 °C with shaking (200 rpm) until its growth reached the exponential growth early phase (OD_600_ = 0.1–0.2). The cells were then centrifuged at 3000× *g* for 10 min. The pellets were resuspended in 2.5 mL LB broth. The isolated phages were added into these suspensions, respectively, according to their optimal MOI and cultured at 37 °C with shaking (200 rpm). Cultures (500 μL) were sampled every 15 min for 2 h. The phage titers at each time point were determined by the double-layer plate method to obtain one-step growth curves of the phages. The assay was performed in triplicate.

### 2.7. DNA Extraction and Identification

Overnight cultures of all phages were harvested for DNA extraction. Chloroform was added to the phage cultures at a final concentration of 0.5%. The cultures were incubated at 37 °C for 1 h to make it lyse completely. Lysates obtained were centrifugated at 8000× *g* for 10 min. The supernatants were collected and then filtered. The filtrates were treated with DNase I (a final concentration of 50 U/mL, Sigma, St. Louis, MO, USA) and RNase A (a final concentration of 50 U/mL, Sigma, St. Louis, MO, USA) at 37 °C for 1 h. Phage particles were treated with 1 mol/L NaCl in ice for 1 h, then concentrated with 10% (*w*/*v*) polyethylene glycol 8000. After centrifuging at 8000× *g* for 15 min, the pellets were collected and resuspended in 1 mL SM buffer (0.05 M Tris-HCl, pH 7.5, 0.1 M NaCl, 0.017 M MgSO_4_, 0.01% gelatin). Phage DNAs were obtained by the phenol chloroform-extraction method, as previously reported [20,21].

The phage genomes were digested with the endonucleases EcoRI, HpaI, RsrII, SacI, Sph I, Bgl II, Sma I, Sac II, SnaB I, Avr II, and Pst I. The products were identified using agarose gel electrophoresis.

### 2.8. Growth Kinetic Curve of In Vitro Lysis

*S. aureus* CVCC 546 was inoculated at a rate of 2% into 20 mL LB broth and cultured at 37 °C with shaking (200 rpm) until its growth reached the exponential growth early phase (OD_600_ = 0.1–0.2). Then, the phages were inoculated into the medium at MOI ratios of 0.01, 0.1, 1, and 10. One milliliter of culture was continuously sampled every 30 min within a period of 10.5 h. The OD_600_ was measured using a spectrophotometer (Thermo Fisher Scientific, Waltham, MA, USA), and growth dynamics curves were constructed based on this value. The assay was performed in triplicate.

### 2.9. Detection of Phages to Remove Biofilms

To explore the ability of phages to remove *S. aureus* biofilms, *S. aureus* CVCC 546 was streaked on LB agar and cultured at 37 °C for 12 h. Then, a single colony was picked and inoculated in a 5 mL LB broth and cultured at 37 °C for 10 h with shaking (200 rpm). The cultures were centrifuged at 12,000× *g* for 10 min. The pellets were collected, and the concentration of the bacteria was adjusted to approximately 1 × 10^8^ CFU/mL with phosphate-buffered saline (PBS). Then, 50 μL of bacterial suspension and 150 μL of LB medium (with a final glucose concentration of 5%) were added to 96-well plates. After treatment at 37 °C for 24 h, the supernatant was discarded. PBS (250 μL) was added to each well, and the wells were washed twice to remove unbound bacteria. Then, 200 μL of phage suspensions (1 × 10^9^ PFU/mL) were added to each well respectively. After treatment for 4, 8, 12, 24, and 36 h, the supernatants in the wells were discarded. The 96-well plates were washed twice and then dried at 60 °C for 10 min. Next, 200 μL of 0.1% crystal violet solution was added to each well and incubated for 10 min. After discarding the crystal violet solution, 250 μL of PBS was added to each well and washed three times to remove the unbound crystal violet. The optical density was measured at 570 nm using a spectrophotometer (Thermo Fisher Scientific, Waltham, MA, USA).

### 2.10. Infectious Inoculum Preparation

A total of 600 μL of *S. aureus* CVCC 546 (1 × 10^8^ CFU/mL) was inoculated into a 30 mL LB broth and cultured at 37 °C overnight. The culture (2 mL) was sampled and centrifuged at 4000× *g* for 15 min. The pellets were collected and washed three times with 1 mL PBS and resuspended in PBS to a concentration of 1 × 10^8^ CFU/mL.

### 2.11. In Vivo Phage Therapy (Mouse Model)

To evaluate the effects of phages against *S. aureus* CVCC 546 in vivo, an experimental *S. aureus*-induced mastitis mouse model was used, as previously described, with some modifications [22,23]. The mice were anesthetized with 10% chloral hydrate with an injection of 0.05 µL/g. The fourth pair of nipples and the surrounding skin were disinfected with 75% alcohol. Then, a 2 mm incision was made using sterilized scissors to expose the mammary duct, and 100 µL of the bacterial suspension (1 × 10^8^ CFU/mL) or sterilized PBS was slowly injected into the mouse mammary duct.

Female BALB/C mice (13-week-old) lactating for seven days were randomly divided into four groups (A–D) with four mice per group. Mice in group A were maintained as healthy mice (blank control). Mice in groups B–D were injected with *S. aureus* CVCC 546 (1 × 10^8^ CFU/mL) to induce mastitis. Two hours after administration, the mice in the B-D groups were treated with 100 µL of PBS, phage 4086-1 (1 × 10^8^ PFU/mL), and ceftiofur sodium (5 mg/kg), respectively. The animals were euthanized, and the mammary tissue samples were collected aseptically.

### 2.12. Bacterial Cell Counting and Detection of Cytokines

The minced mammary glands (100 mg) were homogenized in 1 mL of PBS with TissueLyser II (QIAGEN, Hilden, Germany). The homogenate was serially diluted (1:10) with PBS. Bacterial colonies (CFUs) were counted using the plate method [24].

The homogenate was centrifuged at 4 °C (10,000× *g*) for 10 min. The supernatant was used to measure tumor necrosis factor (TNF-α) and interleukin 6 (IL-6) concentrations. ELISA was performed using a kit (MEIMIAN, Jiangsu, China) according to the manufacturer’s protocol.

### 2.13. Histopathological Analysis

For histological analysis, the dissected mammary glands were fixed in 10% neutral buffered formalin, processed into paraffin wax, and sectioned at a nominal thickness of 5 μm. The slides were stained with hematoxylin and eosin (HE) and morphological changes were analyzed using light microscopy.

### 2.14. Statistical Analysis

Data are presented as mean ± standard deviation (SD). Statistical significance was determined using SPSS 22.0 software (IBM) for Windows, version 22.0. Results within samples were determined using Tukey’s multiple comparison statistical test. Statistical significance was set at *p* < 0.001 or *p* < 0.05.

## 3. Results

### 3.1. Morphology of Phages

**Figure 1:**
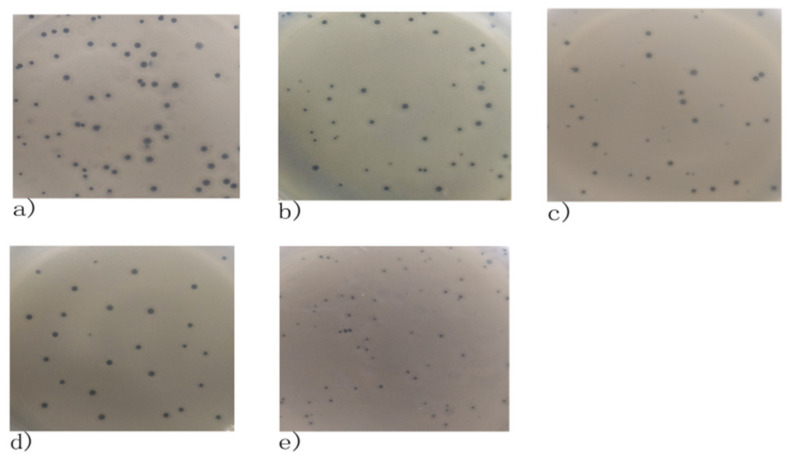
The plaques formed by *S. aureus* phages isolated from clinical mastitis. All the five phages could produce clear plaques. The plaque diameters of 4086-1, 4086-2, 4086-3, 4086-4 were about 2–3 mm, while that of 4086-6 was about 1 mm. (**a**) Phage 4086-1; (**b**) Phage 4086-2; (**c**) Phage 4086-3; (**d**) Phage 4086-4; (**e**) Phage 4086-6.

**Figure 2:**
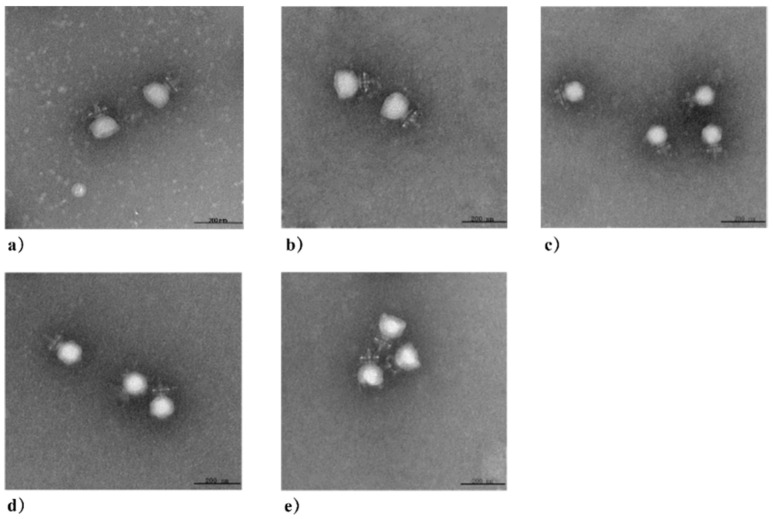
Electron micrograph of *S. aureus* phages isolated from clinical mastitis. All the phages have an isometric head of 37.5 ± 3 nm in diameter, a non-contractile tail with a length of 15 ± 3 nm, and a baseplate structure at the tip of the tail: (**a**) Phage 4086-1; (**b**) Phage 4086-2; (**c**) Phage 4086-3; (**d**) Phage 4086-4; (**e**) Phage 4086-6.

Five phages were isolated and designated as 4086-1, 4086-2, 4086-3, 4086-4, and 4086-6, respectively. All five phages produced clear plaques, indicating that they could lyse *S. aureus* CVCC 546. The plaque diameters of 4086-1, 4086-2, 4086-3, and 4086-4 were all about 2–3 mm, while that of 4086-6 were approximately 1 mm (Figure 1). All the phages had an isometric head of 37.5 ± 3 nm in diameter, a non-contractile tail with a length of 15 ± 3 nm, and a baseplate structure at the tip of the tail (Figure 2). Based on these properties, the five phages should be classified in the family Podoviridae.

### 3.2. Phage Host Range

The host ranges of the five phages were evaluated using a spot test. Phages 4086-1, 4086-2, and 4086-3 could lyse 4 of the 17 strains tested, while phage 4086-4 and 4086-6 could only lyse 2 of 17 strains (Table 1). Overall, the five phages were species-specific, attacking only *S. aureus*. The phages 4086-1, 4086-2, and 4086-3 were found to have a wider host range, which can lyse all four *S. aureus* strains. Phage 4086-4 and phage 4086-6 could only lyse two strains of *S. aureus*.

### 3.3. Optimal Multiplicity of Infection

**Figure 3:**
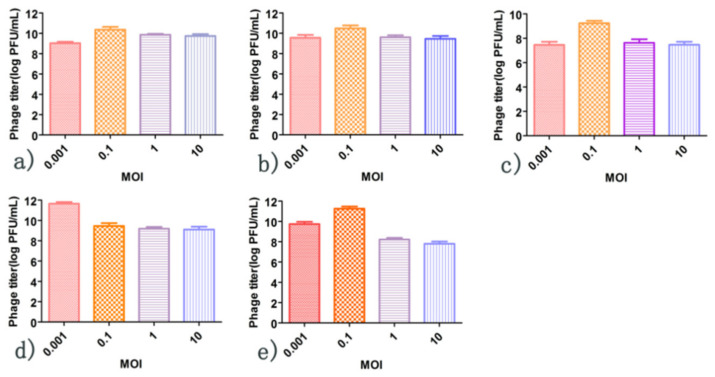
The optimal multiplicity of infection of *S. aureus* phages isolated from clinical mastitis. When the MOI was 0.1, the titers of phages 4086-1, 4086-2, 4086-3, and 4086-6 were the highest, which were 1.46 × 10^10^ PFU/mL, 3.21 × 10^10^ PFU/mL, 7.66 × 10^10^ PFU/mL, and 2.35 × 10^11^ PFU/mL, respectively. While the MOI was 0.001, the titer of phage 4086-4 was the highest at 2.41 × 10^12^ PFU/mL. (**a**) Phage 4086-1; (**b**) Phage 4086-2; (**c**) Phage 4086-3; (**d**) Phage 4086-4; (**e**) Phage 4086-6.

When the MOI was 0.1, the titers of phages 4086-1, 4086-2, 4086-3, and 4086-6 were the highest, which were 1.46 × 10^10^ PFU/mL, 3.21 × 10^10^ PFU/mL, 7.66 × 10^10^ PFU/mL, and 2.35 × 10^11^ PFU/mL, respectively. While the MOI was 0.001, the titer of phage 4086-4 was the highest at 2.41 × 10^12^ PFU/mL (Figure 3).

### 3.4. One-Step Growth Curve

**Figure 4:**
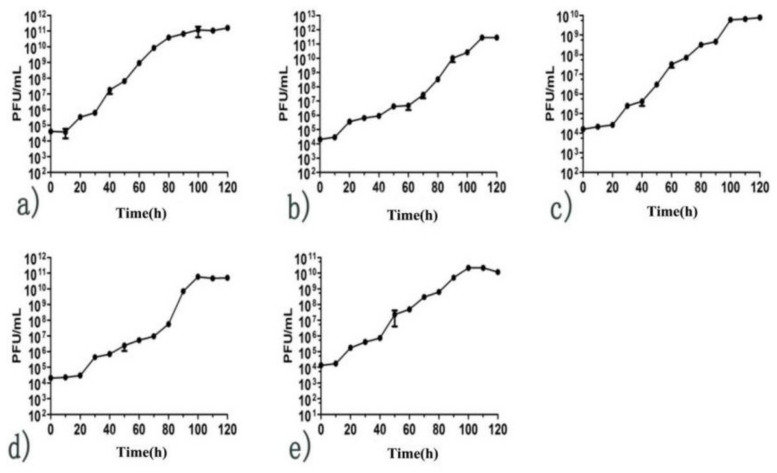
One-step growth curve of *S. aureus* phages isolated from clinical mastitis. The results are shown as the mean ± standard deviation from three independent experiments. The one-step growth curve showed a short latency period (10–20 min) and high burst size (up to 400 PFU/infected cells). (**a**) Phage 4086-1; (**b**) Phage 4086-2; (**c**) Phage 4086-3; (**d**) Phage 4086-4; (**e**) Phage 4086-6.

Based on the MOI of the phages, the one-step growth curve showed a latent period (time interval between the absorption and the start of the first burst) of phages that was approximately 10–20 min. The burst period of the five phages was 80–100 min. Phages 4086-1 and 4086-2 had a larger burst size that was 365.7 PFU/infected cell and 400 PFU/infected cell, respectively (Figure 4).

### 3.5. Phage Genome Identification

**Figure 5:**
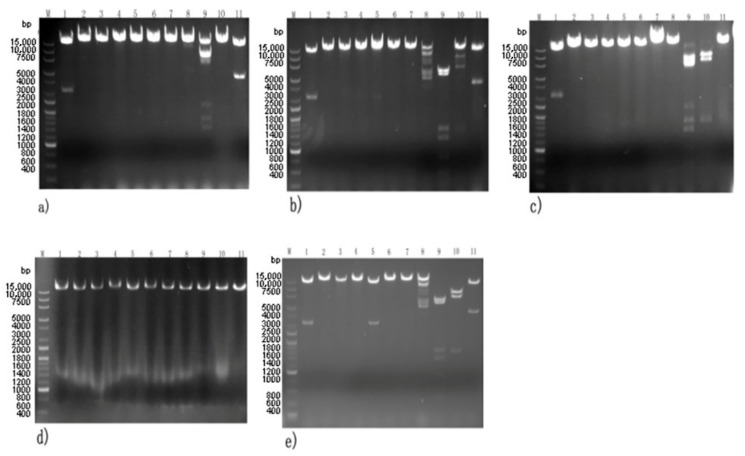
Agarose gel electrophoresis of generated DNA fragments of the isolated phages by digestion with 11 restriction enzymes. Lane M, DL 15,000 marker. Restriction endonuclease digestion map of phage 4086-1 (**a**), phage 4086-2 (**b**), phage 4086-3 (**c**), phage 4086-4 (**d**), phage 4086-6 (**e**) digested by restriction enzymes EcoR I (1), Hpa I (2), Rsr II (3), Sac I (4), Sph I (5), Bgl II (6), Sma I (7), Sac II (8), SnaB I (9), Avr II (10), and Pst I (11), respectively.

Digestion was performed with different endonucleases, and the results are shown in Figure 5. Phage 4086-1 could be digested by EcoRI, SnaBI, and Pst I. Phage 4086-2 could be digested by EcoRI, SacII, SnaB I, Avr II, and Pst I. Phage 4086-3 could be digested by EcoRI, SnaB I, and Avr II. Phage 4086-6 could be digested by EcoRI, SphI, SacII, SnaB I, Avr II, and Pst I. Phage 4086-4 could not be digested by any of these endonucleases. This suggested that phages 4086-1, 4086-2, 4086-3, 4086-4, and 4086-6 were different from each other (Figure 5).

### 3.6. Growth Kinetic Curve of In Vitro Lysis

**Figure 6:**
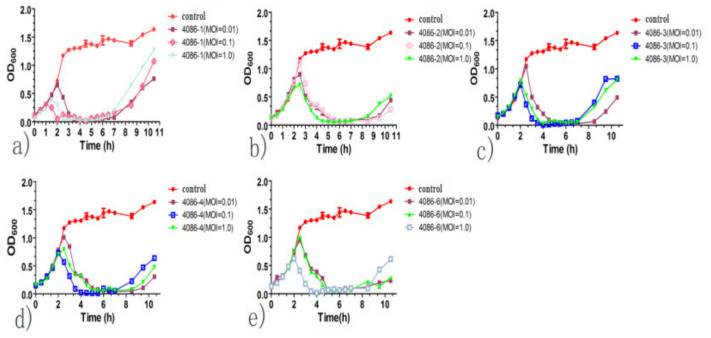
Effect of the isolated phages on bacterial growth. Host bacteria *S. aureus* CVCC 546 were infected with phage 4086-1, phage 4086-2, phage 4086-3, phage 4086-4, and phage 4086-6 to determine the OD600 at different times. Phages could effectively inhibit the growth of *S. aureus* CVCC 546 when the MOI was 0.01, 0.1, 1, and 10. (**a**) Phage 4086-1; (**b**) Phage 4086-2; (**c**) Phage 4086-3; (**d**) Phage 4086-4; (**e**) Phage 4086-6.

When *S. aureus* CVCC 546 was inoculated and cultured to the exponential growth early phase, the phages were inoculated at an MOI of 0.01, 0.1, 1, and 10. Two hours after infection, the cultures showed a significant decrease in OD_600_ compared to that of the control. The results showed that the phages effectively inhibited the growth of *S. aureus* CVCC 546. After 8 h of treatment, phage-resistant bacteria appeared (Figure 6).

### 3.7. The Ability of Phages to Remove Biofilms

**Figure 7:**
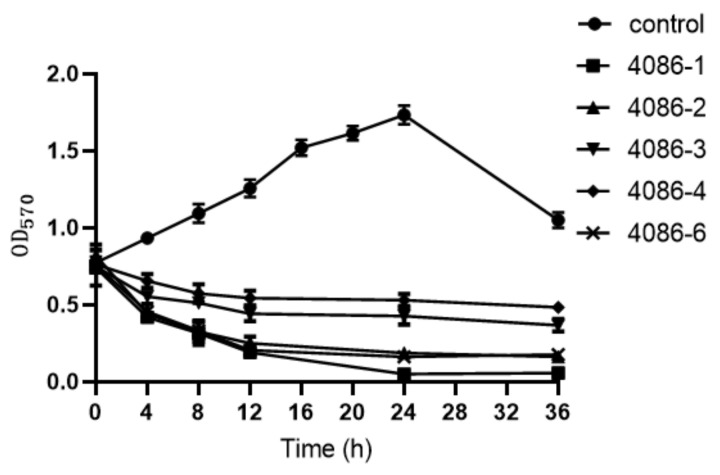
The effects of the isolated phages on the removal of the *S. aureus* biofilms. Bacterial biofilm formation was measured at various time points.

The effects of phages on the removal of the *S. aureus* biofilms were investigated. Bacterial biofilm formation was measured at various time points. The extent of an incremental decrease in biomass in the biofilms was characterized by the OD_570_ value after crystal violet staining and extraction of the dye with ethanol. The biofilms decreased effectively after 24 h of treatment (Figure 7). In particular, phages 4086-1, 4086-2, and 4086-6 had stronger abilities to remove biofilms.

### 3.8. Colony-Forming Units

**Figure 8:**
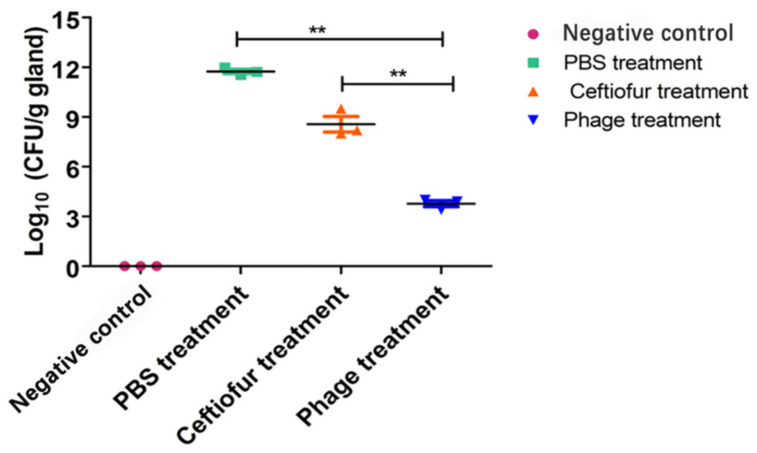
*S. aureus* CFUs in the mammary glands after phage treatment. Each point on the graph corresponds to the log_10_ CFU/g value of an individual gland. The bar represents the median value for each group. **, *p* < 0.01.

The *S. aureus* counts of the infected mammary glands were measured using the plate-counting method. *S. aureus* CFUs were greatly reduced after phage treatment (Figure 8), indicating that phages could effectively reduce the number of *S. aureus*.

### 3.9. Changes in TNF-α and IL-6 Concentrations

**Figure 9:**
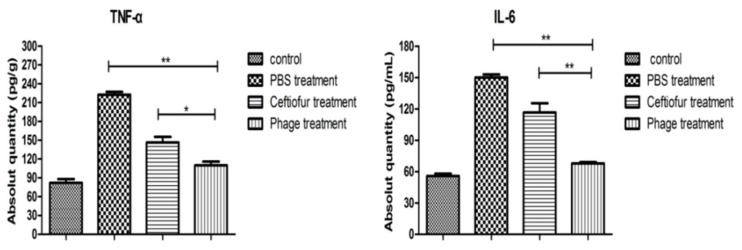
Concentration of TNF-α and IL-6 after phage treatment using an induced mastitis model in mice. The concentrations of IL-6 and TNF-α in mice mammary tissues decreased significantly. The results are reported as the mean titer ± SD for four mice per group. *, *p* < 0.05; **, *p* < 0.01.

The infected mammary glands were treated with the phage 4086-1. The concentrations of TNF-α and IL-6 were determined using ELISA kits. Compared with the PBS-treated group, the ceftiofur sodium-treatment group and the phage-treatment group both showed significant decreases in the concentrations of TNF-α and IL-6. The phage-treated group displayed the most significant reduction in the concentrations of TNF-α and IL-6 (*p* < 0.01) (Figure 9). The above results demonstrated that phages could effectively alleviate inflammatory responses after phage treatment.

### 3.10. Histopathological Analysis

**Figure 10:**
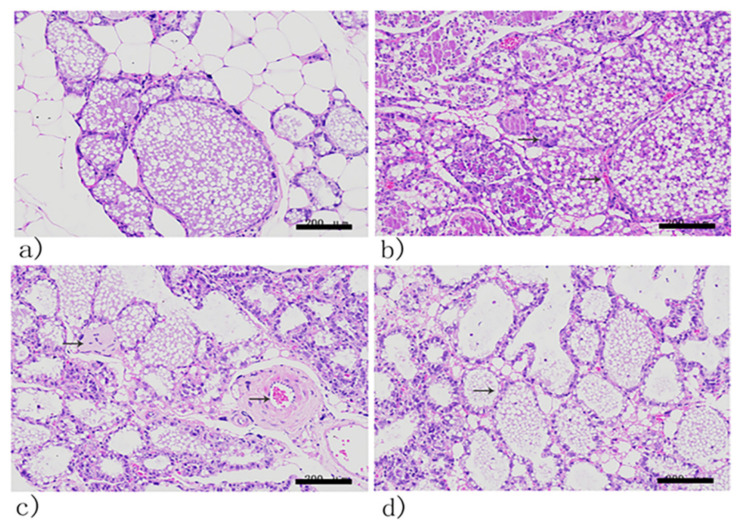
Histopathological sections of mammary glands after phage treatment. (**a**) Tissue sections of the mammary glands of the control group revealed no abnormal histopathological changes. (**b**) In the PBS-treatment group, the acinar epithelial cells were partially necrotic and apoptotic, with a large number of inflammatory cells infiltrating the acinar space and acini (as indicated by the arrows). (**c**) The mammary glands of the ceftiofur sodium-treated groups showed relatively fewer inflammatory cells infiltrating (as indicated by the arrows). (**d**) Mice treated with phages exhibited normal mammary glands. The cuboidal epithelial cells and acini were intact (as indicated by the arrow). Sections were stained with hematoxylin and eosin and photographed at 40× magnification.

Histopathological analysis of mammary glands is shown in Figure 8. Tissue sections of the mammary glands of healthy mice revealed no abnormal histopathological changes (Figure 10a). In contrast, a massive proliferation of bacteria could be observed in the PBS-treated group. The acinar epithelial cells were partially necrotic and apoptotic, and inflammatory infiltrates were observed around the acinar space and acini (Figure 10b).

The mammary glands of the ceftiofur sodium-treated groups showed relatively fewer pathological changes. Mammary epithelial tissues were relatively intact with a variable, minimal to moderate degree of necrotic (or apoptotic) epithelial cells and a small amount of inflammatory cell infiltration (Figure 10c). Mice treated with phages display normal mammary glands. The cuboidal epithelial cells and acini were intact (Figure 10d). Furthermore, inflammatory cell infiltration was reduced.

## 4. Discussion

In the present study, five lytic phages were isolated from milk samples secreted by mastitis cows and characterized. After characterization of the phages in vitro, a mouse model for staphylococcal mastitis was established to evaluate the effect of the phage 4086-1 in vivo. The results showed that the phage 4086-1 was a promising therapeutic antibacterial agent as it exhibited an outstanding efficacy for treating *S. aureus*-induced mastitis.

Taxonomically, most *S. aureus* phages belong to the Siphoviridae family, and only a few belong to the Podoviridae and Myoviridae families, which are mainly virulent phages [25]. The five phages isolated in this experiment belonged to the Podoviridae family and presented favorable characteristics, such as species-specificity, short latent periods, and large burst sizes. Especially, the burst sizes of phages 4086-1 and 4086-2 are 365.7 and 400 PFU/infected cell, respectively. The burst sizes were much larger than that of phage CSA13 (230 PFU/infected cell) and phage LM12 (52 PFU/infected cell) isolated by Yoyeon Cha [26] and Joana Barros [27].

Many antibiotic-resistant bacteria can form biofilms, which can protect the bacteria in the membrane from physical, chemical, and biological factors. Once the biofilm has formed, the bacteria can evade the host immune response and limit the therapeutic effect of antibiotics. The formation of biofilm causes the infection to alternate between the static and acute stages, which is difficult to cure [28]. Therefore, the search for methods that can suppress biofilm formation is an immensely important and relevant issue. Many studies have shown that phages have significant effects on biofilm removal. Phages phiIBB-PAA2 and phiIBB-PAP21 could both reduce the *Pseudomonas aeruginosa* biofilm population by approximately 1–2 log after 2 h of infection, and the reduction was further enhanced after 6 h [29]. It has been reported that a single phage or phage cocktail has a good therapeutic effect on biofilm formation by *E. coli*, *A. baumannii*, and *P. aeruginosa* in the chronically infected skin of pigs, highlighting its potential as an alternative or complementary treatment agent to control wound infections [30]. Many strains of *S. aureus* can form biofilms during growth and reproduction, making the infection more difficult to cure. In this study, five isolated phages were used to remove biofilms formed by *S. aureus* CVCC 546. Bacterial biofilm formation was measured at different time points after treatment with the isolated phages. The amount of biofilm decreased effectively after 24 h of treatment. In particular, phages 4086-1, 4086-2, and 4086-6 had stronger abilities to remove biofilms.

*S. aureus* is one of the major causes of mastitis. Most of the *S. aureus* mastitis isolates displayed antibiotic insensitivity in vitro [6], and they are more difficult to eliminate in vivo with the established clinical treatments [31,32]. Moreover, it has been reported that multiple multidrug-resistant strains present a serious challenge in the treatment of *S. aureus*-induced mastitis infections [33,34]. This study evaluated the efficacy of the *S. aureus* phage 4086-1 in treating mastitis using mouse models.

Phage therapy studies with mouse models have shown that the phage 4086-1 was efficient in reducing the amount of *S. aureus* in the mammary glands over time. Increased concentrations of TNF-α and IL-6 were detected in mice following the induction of mastitis, which indicated an ongoing inflammatory process. Similarly, after treatment with phages, the concentrations of TNF-α and IL-6 in the mammary glands decreased significantly. Both the cytokines could be involved in cell proliferation, inflammation, and immunity at the local and systemic concentrations and were often used to assess the degree of inflammation in mouse mammary glands [35,36]. The results showed that phages could effectively alleviate humoral and cell-mediated immune responses. It has also been reported that the concentrations of TNF-α and IL-6 could be downregulated after treatment with a single-dose T4 phage in an *E. coli*-induced mastitis mouse model [2]. This indicated that *E. coli* phages could effectively alleviate inflammatory responses, which corresponds to the results described in this study.

In addition, the pathological analysis also showed that the mammary glands in the phage-treatment groups were relatively intact, with significantly less inflammatory cell infiltration. This demonstrated that phages were efficient in reducing the density of the infecting bacterial population to a level that may allow the host immune response to defend and clear the infections, which provides evidence for the application of phage therapy for the treatment of mastitis.

In summary, the therapeutic effects of *S. aureus* phages on mastitis caused by MRSA strains were evaluated. Based on the data obtained, phage therapy could be considered an innovative approach to replace antibiotics in the treatment of drug-resistant strains.

## 5. Conclusions

In this study, we isolated five lytic phages from the milk of cows suffering from mastitis, which could lyse *S. aureus* CVCC 546 (MRSA strain) and belong to the family Podoviridae. The one-step growth curve showed a short latency period (10–20 min) and high burst size (up to 400 PFU/infected cell). In a mouse model of mastitis, phage treatment effectively alleviated inflammatory responses and significantly reduced inflammatory infiltration in mammary glands. These results demonstrated that phage treatment may be a potential therapy against mastitis caused by drug-resistant *S. aureus*.

## Figures and Tables

**Table 1:** Host range of *S. aureus* phages isolated from clinical mastitis by spot test.

	Phage 4086-1	Phage 4086-2	Phage 4086-3	Phage 4086-4	Phage 4086-6
*S. aureus* CVCC 546	+	+	+	+	+
*S. aureus* 4086	+	+	+	+	+
*S. aureus* Ben	+	+	+	−	−
*S. aureus* ATCC 43,300	+	+	+	−	−
*Micrococcus luteus* 26,003	−	−	−	−	−
*Staphylococcus saparophytics* 17	−	−	−	−	−
*Staphylococcus saparophytics* E4	−	−	−	−	−
*Staphylococcus saparophytics* X4	−	−	−	−	−
*Staphylococcus haemolyticus* 13	−	−	−	−	−
*Staphylococcus xylosus* 17	−	−	−	−	−
*Enterococcus faecalis* 13	−	−	−	−	−
*Bacillus subtilis*	−	−	−	−	−
*Pasteurella* p−1059	−	−	−	−	−
*Staphylococcus gallinarum* CVCC 528	−	−	−	−	−
*Escherichia coli* BL 21	−	−	−	−	−
*Escherichia coli* DH5α	−	−	−	−	−
*Escherichia coli* CVCC 10,141	−	−	−	−	−
Total	4	4	4	2	2

(+) sensitive (clear plaque); (−): insensitive (no plaque).

## Data Availability

De-identified data are available through personal email requests to the authors, Fei Teng (teng1085579571@163.com) and Xinyuan Qiao (qiaoxinyuan@126.com).
