# Correction: Teng et al. Efficacy Assessment of Phage Therapy in Treating *Staphylococcus aureus*-Induced Mastitis in Mice. *Viruses* 2022, *14*, 620

**DOI:** 10.3390/v16030319

**Published:** 2024-02-20

**Authors:** Fei Teng, Xiaoyu Xiong, Songsong Zhang, Guiwei Li, Ruichong Wang, Lanlan Zhang, Xiaona Wang, Han Zhou, Jiaxuan Li, Yijing Li, Yanping Jiang, Wen Cui, Lijie Tang, Li Wang, Xinyuan Qiao

**Affiliations:** 1 Heilongjiang Key Laboratory for Animal Disease Control and Pharmaceutical Development, Department of Preventive, Veterinary Medicine, College of Veterinary Medicine, Northeast Agricultural University, Harbin 150038, China; teng1085579571@163.com (F.T.); sum670238789@163.com (X.X.); s15130036557@126.com (S.Z.); xiaonawang0319@163.com (X.W.); zhouhan9659@163.com (H.Z.); lijiaxuan.1993@163.com (J.L.); yijingli@163.com (Y.L.); jiangyanping2017@126.com (Y.J.); cuiwen_200@163.com (W.C.); tanglijie@neau.edu.cn (L.T.); 2 Branch of Animal Husbandry and Veterinary of Heilongjiang Academy of Agricultural Sciences, Qiqihar 161000, China; hljslgyxh@163.com; 3 Department for Radiological Protection, Heilongjiang Province Center for Disease Control and Prevention, Harbin 150030, China; mice4@126.com; 4 Promotion Demonstration Department of Heilongjiang Fishery Technology Extension Station, Harbin 150030, China; zlllgw@163.com

In the original publication [1], there was a mistake in Figure 2 as published. Panel 2a of Figure 2 had been processed to hide an uneven background for better observation. In this correction, a new image of Phage 4086-1 is provided to replace panel 2a. Corrected Figure 2 appears below.

The authors state that the scientific conclusions of the article are unaffected. This correction was approved by the Academic Editor. The original publication has also been updated.

## Figures and Tables

**Figure 2:**
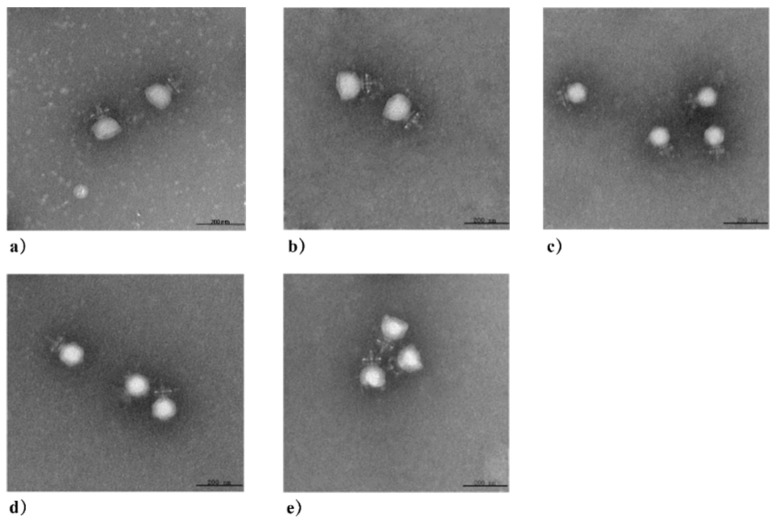
Electron micrograph of *S. aureus* phages isolated from clinical mastitis. All the phages have an isometric head of 37.5 ± 3 nm in diameter, a non-contractile tail with a length of 15 ± 3 nm, and a baseplate structure at the tip of the tail: (**a**) Phage 4086-1; (**b**) Phage 4086-2; (**c**) Phage 4086-3; (**d**) Phage 4086-4; (**e**) Phage 4086-6.
